# Identifying proteomic risk factors for overall, aggressive, and early onset prostate cancer using Mendelian Randomisation and tumour spatial transcriptomics

**DOI:** 10.1016/j.ebiom.2024.105168

**Published:** 2024-06-14

**Authors:** Trishna A. Desai, Åsa K. Hedman, Marios Dimitriou, Mine Koprulu, Sandy Figiel, Wencheng Yin, Mattias Johansson, Eleanor L. Watts, Joshua R. Atkins, Aleksandr V. Sokolov, Helgi B. Schiöth, Marc J. Gunter, Konstantinos K. Tsilidis, Richard M. Martin, Maik Pietzner, Claudia Langenberg, Ian G. Mills, Alastair D. Lamb, Anders Mälarstig, Tim J. Key, The PRACTICAL Consortium, Ruth C. Travis, Karl Smith-Byrne

**Affiliations:** a Cancer Epidemiology Unit, Oxford Population Health, University of Oxford, Oxford, United Kingdom; b External Science and Innovation, Pfizer Worldwide Research, Development and Medical, Stockholm, Sweden; c Department of Medical Epidemiology and Biostatistics, Karolinska Institutet, Stockholm, Sweden; d MRC Epidemiology Unit, University of Cambridge, United Kingdom; e University of Oxford, Nuffield Department of Surgical Sciences, Oxford, United Kingdom; f Genomic Epidemiology Branch, International Agency for Research on Cancer (IARC-WHO), Lyon, France; g Metabolic Epidemiology Branch, Division of Cancer Epidemiology and Genetics, National Cancer Institute, Rockville, MD, USA; h Department of Surgical Sciences, Functional Pharmacology and Neuroscience Uppsala University, 75124, Uppsala, Sweden; i Department of Epidemiology and Biostatistics, School of Public Health, Imperial College London, St Mary's Campus, Norfolk Place, London, W2 1PG, United Kingdom; j Department of Hygiene and Epidemiology, University of Ioannina School of Medicine, Ioannina, Greece; k Population Health Sciences, Bristol Medical School, University of Bristol, Bristol, United Kingdom; l MRC Integrative Epidemiology Unit, University of Bristol, Bristol, United Kingdom; m NIHR Bristol Biomedical Research Centre, Hospitals Bristol and Weston NHS Foundation Trust and the University of Bristol, Bristol, United Kingdom; n Computational Medicine, Berlin Institute of HealthHealth (BIH) at Charité - Univeritätsmedizin– Universitätsmedizin Berlin, Berlin, Germany; o Precision Healthcare University Research Institute, Queen Mary University of London, London, United Kingdom

**Keywords:** Proteins, Proteomics, -Omics, Mendelian randomisation, Cancer, Genetic epidemiology, Spatial transcriptomics

## Abstract

**Background:**

Understanding the role of circulating proteins in prostate cancer risk can reveal key biological pathways and identify novel targets for cancer prevention.

**Methods:**

We investigated the association of 2002 genetically predicted circulating protein levels with risk of prostate cancer overall, and of aggressive and early onset disease, using *cis-*pQTL Mendelian randomisation (MR) and colocalisation. Findings for proteins with support from both MR, after correction for multiple-testing, and colocalisation were replicated using two independent cancer GWAS, one of European and one of African ancestry. Proteins with evidence of prostate-specific tissue expression were additionally investigated using spatial transcriptomic data in prostate tumour tissue to assess their role in tumour aggressiveness. Finally, we mapped risk proteins to drug and ongoing clinical trials targets.

**Findings:**

We identified 20 proteins genetically linked to prostate cancer risk (14 for overall [8 specific], 7 for aggressive [3 specific], and 8 for early onset disease [2 specific]), of which the majority replicated where data were available. Among these were proteins associated with aggressive disease, such as PPA2 [Odds Ratio (OR) per 1 SD increment = 2.13, 95% CI: 1.54–2.93], PYY [OR = 1.87, 95% CI: 1.43–2.44] and PRSS3 [OR = 0.80, 95% CI: 0.73–0.89], and those associated with early onset disease, including EHPB1 [OR = 2.89, 95% CI: 1.99–4.21], POGLUT3 [OR = 0.76, 95% CI: 0.67–0.86] and TPM3 [OR = 0.47, 95% CI: 0.34–0.64]. We confirmed an inverse association of MSMB with prostate cancer overall [OR = 0.81, 95% CI: 0.80–0.82], and also found an inverse association with both aggressive [OR = 0.84, 95% CI: 0.82–0.86] and early onset disease [OR = 0.71, 95% CI: 0.68–0.74]. Using spatial transcriptomics data, we identified MSMB as the genome-wide top-most predictive gene to distinguish benign regions from high grade cancer regions that comparatively had five-fold lower MSMB expression. Additionally, ten proteins that were associated with prostate cancer risk also mapped to existing therapeutic interventions.

**Interpretation:**

Our findings emphasise the importance of proteomics for improving our understanding of prostate cancer aetiology and of opportunities for novel therapeutic interventions. Additionally, we demonstrate the added benefit of in-depth functional analyses to triangulate the role of risk proteins in the clinical aggressiveness of prostate tumours. Using these integrated methods, we identify a subset of risk proteins associated with aggressive and early onset disease as priorities for investigation for the future prevention and treatment of prostate cancer.

**Funding:**

This work was supported by 10.13039/501100000289Cancer Research UK (grant no. C8221/A29017).

**Research in context**

**Evidence before this study**

Prostate cancer is the second most commonly diagnosed cancer among men worldwide with poor prognosis for men diagnosed with aggressive forms, however few established risk factors for the disease have been confirmed. Both pre-clinical molecular research and prospective epidemiological studies have suggested individual proteins may be important for the aetiology of prostate cancer, including insulin growth factor-I and microsmeinoprotein-beta. However, the role of most proteins in relation to prostate cancer risk remains unknown, largely due to limitations of proteomic measurements in large-scale population studies. Recent advances in multiplexed platforms for proteomics and large genetic consortia for cancer outcomes now allow for a more comprehensive analysis of endogenous protein levels and risk for prostate cancer.

**Added value of this study**

Using a large dataset on genetically predicted prostate cancer, we employed a Mendelian randomisation analysis and colocalisation pipeline to investigate up to 2002 proteins in relation to the risk of prostate cancer overall (n = 85,554 cases and 91,972 controls), and for aggressive (n = 15,167 cases and 53,308 controls) and early onset disease (n = 6988 cases and 44,256 controls). We found 20 proteins to be associated with at least one prostate cancer outcome, and were able to replicated these findings in independent European and African-ancestry populations, where data existed. We additionally reported that half of these proteins map to therapeutic agents that are currently being investigated for cancer and non-cancer endpoints. Finally, using spatial transcriptomics, we showed a directionally concordant relationship in the tissue for the protective effects of MSMB, the protein most strongly associated with all three prostate cancer outcomes, by demonstrating lower expression of MSMB in higher grade prostate cancer tumour tissue compared to lower grade and benign regions.

**Implications of all the available evidence**

Our proteogenomic analyses have identified a suite of proteins that may have a role in prostate cancer aetiology, including some that may be important for the development of aggressive and early onset disease, and several that are already targets for therapeutic interventions. We also demonstrate the value of layering genetic population-based methodology with tissue-based evidence to further elucidate prostate cancer aetiology. These integrated approaches aid in prioritising subsets of proteins for further evaluation as aetiological biomarkers which may ultimately inform cancer prevention strategies.

## Introduction

Prostate cancer is a heterogeneous disease with a high survival rate for those diagnosed with indolent or low-stage disease, but a less than 50% 5-year survival rate for those diagnosed with aggressive or metastatic cancer.^1^ The proportion of these clinically aggressive cases is higher among men younger than 55 years (early onset disease), which contributes to premature death among these men.^2^^,^^3^ However, few risk factors for prostate cancer have been established. These include: advanced age, African ancestry, family history of the disease, circulating levels of insulin-like growth factor I and microseminoprotein-beta (MSMB), with little evidence for successful strategies for prevention.^4^, ^5^, ^6^, ^7^

Recent advances in multiplexed and high throughput platforms as well as the widespread availability of genotypic arrays have identified genetic variants that determine circulating levels of thousands of proteins, known as protein-quantitative trait loci (pQTL). PQTL, in particular those lying in or near a protein's cognate gene (referred to as *cis*-pQTL), can be leveraged to identify candidate aetiological proteins for cancer risk through Mendelian randomisation (MR) analyses, an approach that can limit the impact of reverse causality when relevant assumptions are met.^8^^,^^9^ In the context of *cis*-pQTL, these assumptions require that the *cis*-pQTL is robustly associated with levels of its encoding protein (relevance assumption), that the *cis*-pQTL is associated with the outcome of interest only through the association with its encoding protein (exclusion restriction assumption), and that the *cis*-pQTL shares no common causes with the outcome (independence assumption).^10^ MR can also be complemented with colocalisation analyses to further exclude confounding by linkage disequilibrium (LD).^11^ Candidate aetiological proteins for cancer risk identified using these methods can provide a valuable starting point for further analyses using more resource-intense methods, such as spatial transcriptomics, where their functional importance at the tissue level can be directly interrogated to triangulate their role in aetiology.^12^^,^^13^

Using an integrated *cis-*pQTL MR and colocalisation pipeline, we analysed the associations of 2002 unique proteins with overall, aggressive, and early onset prostate cancer and replicated and mapped those with significant findings to drug targets. Additionally, we investigated the spatial distribution and gene expression profiles of a subset of these proteins in prostate tumour tissue using spatial transcriptomics. In doing so, we demonstrate the value of protein MR and colocalisation analyses to identify proteins that may have a causal role in the tumour aggressiveness.

## Methods

### Overall study design

We extracted *cis* genetic instruments for circulating protein levels from publicly available datasets, and harmonised these *cis*-pQTL with the GWAS results from an international prostate cancer consortium, including aggressive and early onset subtypes (Supplementary Figure S1). We subsequently estimated risk associations for protein levels using *cis*-pQTL MR against each of these three prostate cancer endpoints. All associations passing a multiple testing threshold in MR analyses were then followed up with colocalisation analyses. Where data were available, we performed replication analyses in an external prostate cancer GWAS in European and African ancestry (using African ancestry specific *cis*-pQTLs—see below) populations for proteins with evidence from MR and colocalisation analyses. For proteins identified as risk factors for prostate cancer with evidence of specific expression in the prostate tissue, we additionally performed analyses using spatial transcriptomics to gain insights into the spatial distribution and gene expression patterns of these proteins in prostate tumour samples. Finally, we conducted an exploratory analysis restricting to *cis-*pQTL whose cognate genes are established drug targets.

### Identification of cis-pQTL

Genetic instruments for *cis*-pQTL were extracted from 4 publicly available protein GWAS at p < 5 × 10^−8^ and clumped at R^2^ = 0.01 within their originating panel (instruments and study source characteristics presented in Supplementary Tables S1 and S2).^14^, ^15^, ^16^, ^17^ *Cis*-instruments were defined in the first instance as those that were genome-wide significant (p < 5 × 10^−8^) within 1 Mb of the transcription start side of the measured protein encoded gene, or as the sentinel *cis*-pQTL for the measured protein, depending on data availability. We additionally gathered data on *cis-*pQTL from published GWAS present on the OpenGWAS platform using a relaxed p-value threshold of 5 × 10^−5^ due to the high biological plausibility of identifying *cis-*pQTL at or near a protein's cognate gene.^18^^,^^19^ Specifically, we extracted unreported *cis*-pQTL from the genomic region 1 megabase up and downstream of the cognate gene for a given protein GWAS (Supplementary Table S2). We subsequently extracted all instruments where no *cis*-pQTL was present at p < 5 × 10^−8^ but at least one *cis*-pQTL was present at p < 5 × 10^−05^.

All instruments were mapped to Uniprot IDs, and *cis-*pQTL with weak instrument strength at F_stat_ <10 [β^2^/σ^2^] were excluded from the study in order to mitigate weak instrument bias. For *cis*-pQTL that were not present in the cancer outcome data, SNP proxies were selected at r^2^_max_ where r^2^ > 0.8 in 1000 genomes CEU population with the index *cis-*pQTL. To ensure independence of instruments, SNPs were clumped at r^2^ = 0.01 within each study source using the PLINK clumping method. In total, 2002 unique plasma proteins that fit these criteria were included in analyses.

### Cancer outcome data

Genetic associations for overall, aggressive, and early onset prostate cancer were obtained from the Prostate Cancer Association Group to Investigate Cancer Associated Alterations in the Genome (PRACTICAL) consortium (Supplementary material).^20^ Full study characteristics have been described previously, but briefly, summary statistics for SNP associations with prostate cancer and subtypes were generated from the PRACTICAL consortium using 85,554 overall prostate cancer cases and 91,972 controls (database of Genotypes and Phenotypes [dbGaP] project #31553), 15,167 aggressive prostate cancer cases and 58,308 controls, and 6988 cases of early onset disease and 44,256 controls, all of European ancestry.^21^^,^^22^ Aggressive prostate cancer is defined in PRACTICAL as cases having metastatic disease or Gleason score ≥8 or PSA >100 ng/mL or prostate cancer death. Early onset prostate cancer cases are defined as those diagnosed before the age of 55 years. Genotype information was imputed for samples using the 2014 release of the 1000 Genomes Project as a reference panel. Based on the median proportion of variance explained for *cis*-pQTL used in this study (0.01), we had 80% power to detect an odds ratio of 1.14 for overall prostate cancer, 1.29 for aggressive disease and 1.36 for early onset disease.

### Statistics

#### Two-sample Mendelian randomisation

*Cis-*pQTL data were harmonised using the *TwoSampleMR* package and matched to each cancer outcome by rsID and by matching on effect and other allele, and oriented to the protein-increasing allele. All GWAS were on the hg19 build and there was no participant overlap between the protein GWAS and the cancer GWAS. Two-sample Mendelian randomisation (MR) was subsequently performed for each *cis*-pQTL on risk of overall, aggressive, and early onset prostate cancer using the Wald-ratio method (*β*_*cancer/*_*β*_*protein*_). Resulting associations where the *p*_Wald_ passed a Bonferroni-corrected threshold of significance based on the total number of unique proteins assessed for each of the three prostate cancer outcomes were taken forward in analyses (*p*_Wald_ < 0.05/N_Proteins analysed_ _per cancer outcome_).^23^ Multiple independent [*r*^2^ < 0.01] *cis*-pQTL that proxied the same protein and both associated after correction for multiple testing and colocalised with the same prostate cancer outcome, were combined using the inverse-variance weighted method (IVW). Odds ratio estimates are scaled per standard deviation increment in relative and normalised circulating protein concentrations.

### Colocalisation

Colocalisation analyses were performed to assess the probability that the protein and cancer instruments share a causal variant, fulfilling the important exchangeability assumption of MR.^9^^,^^11^ Specifically, single and conditional iterative colocalisation analyses were performed for all *cis-*pQTL MR results that passed a Bonferroni correction for multiple testing based on the number of unique proteins in the study (p < 0.05/N _Proteins_), using all variants within a 75 kb region up- and downstream from the index *cis-*pQTL to assess confounding by linkage disequilibrium.^11^^,^^24^ To mitigate the chance of false-positive findings, we selected priors of P1: 1 × 10^−3^, P2: 1 × 10^−4^, and P12: 1 × 10^−5^, which roughly equate to a 0.1% prior belief in colocalisation (PP4).^25^ We defined a threshold PP4 in support of a shared association for a protein and cancer signal at 0.70 to take proteins forward for subsequent analysis and the highest PP4 of any method of colocalisation was recorded to assess confidence in the shared association for each SNP assessed.

### Replication of robust proteins in European and African ancestry populations

We conducted a replication analysis of *cis*-pQTL MR associations passing both multiple testing correction and colocalisation threshold (referred to as *robust* proteins) using an external GWAS in a European ancestry population of overall prostate cancer risk. GWAS summary statistics represented a meta-analysis in FinnGen r9 and the UK Biobank (20,907 cases & 289,710 controls).^26^^,^^27^ Additionally, where possible, we also performed replication analyses using *cis-*pQTL identified in an African ancestry protein GWAS in the Atherosclerosis Risk in Communities study (4657 proteins in 1871 African-ancestry participants) and a GWAS of overall prostate cancer among African-ancestry populations obtained from dbGaP (project #31553) containing data from the AAPC GWAS, Ghana Prostate Study, ProHealth Kaiser GWAS, and ELLIPSE OncoArray (10,368 cases and 10,986 controls).^22^^,^^28^ GWAS for aggressive and early onset prostate cancer were unavailable to use as a replicate sample in either ancestry population. We considered a directionally concordant risk estimates and Wald ratio p < 0.05 using external data to positively indicate replication. No sample overlap was present between samples used to generate protein associations and those used to conduct replication analyses.

### Drug target pQTL analyses

We restricted our MR results to those *cis*-pQTL that share a cognate gene that is an established drug target by reference to the DrugBank, Therapeutic Target Database, Pharos consortium, ClinicalTrials.gov or expert curation.^29^, ^30^, ^31^ As above, we defined *robust* associations as Wald p < 0.05/N_Proteins_, where N_Proteins_ is the number of unique proteins analysed for a given cancer outcome that were identified as the cognate gene of a pharmaceutical target and PP4 >0.7. Additionally, all proteins identified in overall and drug target analyses were queried in the Cortellis database (https://www.cortellis.com) to assess the highest current level of clinical development stage.

Statistical analyses were performed in R version 4.1 and all tests of significance were two-sided, where p-values < 0.05 were considered statistically significant. MR analyses were performed using the *TwoSampleMR* R package and colocalisation analyses were performed using the *coloc* R package.^18^^,^^24^

### Gene expression analysis using spatial transcriptomics

Spatial transcriptomics provides a spatial map of gene expression within the target tissue. This spatial information can be used to investigate the variation in gene expression by healthy tissue and tumour type intratumourally, and as a result, it can provide valuable insights into tumourigenesis and inform causal inference in this molecularly heterogeneous disease.^32^^,^^33^ Spatial transcriptomic analysis was performed for those proteins that passed both the multiple testing correction and colocalisation threshold, and that also showed high expression in the prostate epithelium.^34^ Data for spatial transcriptomics were obtained from our previously published dataset derived from radical prostatectomy tissue taken from a patient with multifocal prostate cancer.^35^ Our analysis focused on eight distinct tissue sections, which collectively comprised of 32,156 spots, some of which contained regions of cancer as well as histo-pathologically benign prostate tissue, and some of which did not contain cancer. To ensure data quality, samples with less than 500 Unique Molecular Identifier (UMI) counts were excluded from the analysis. The initial fastq files were processed using the 10× Visium Spaceranger software. Spaceranger outputs were then aggregated and normalized (using *aggr*) to correct for batch effects, enabling the conversion of the files into comparable gene expression data and ensuring variance reduction between samples and within cells. A consensus pathology approach was employed involving two pathologists who independently annotated each spatial transcriptomics spot, with the aim to include those that predominantly contained epithelial cells, which comprised approximately 1–15 cells. Violin plots were generated using Graphpad Prism (version 10).

### Iterative random forest network using spatial transcriptomics

We used the iterative random forest (iRF) method to identify the most robust gene expression network discriminating between benign and highest Gleason grade group histology, after normalising data using SCTransform.^36^ The criteria we used to select a credible random forest model was stability >0.8 and precision >0.8 and the resulting gene network was visualised using Gephi 0.99.

### Ethics statement

Genetic analyses were performed using summary statistics and did not require individual level data. All studies contributing to these analyses obtained ethics approval from institutional review boards in their country and all participants included in underlying data provided informed consent.

### Role of funders

The funding institutions had no role in the design and conduct of the study including data collection, analysis, and interpretation of results, or the preparation, review and decision to submit the manuscript for publication.

## Results

**Fig. 1:**
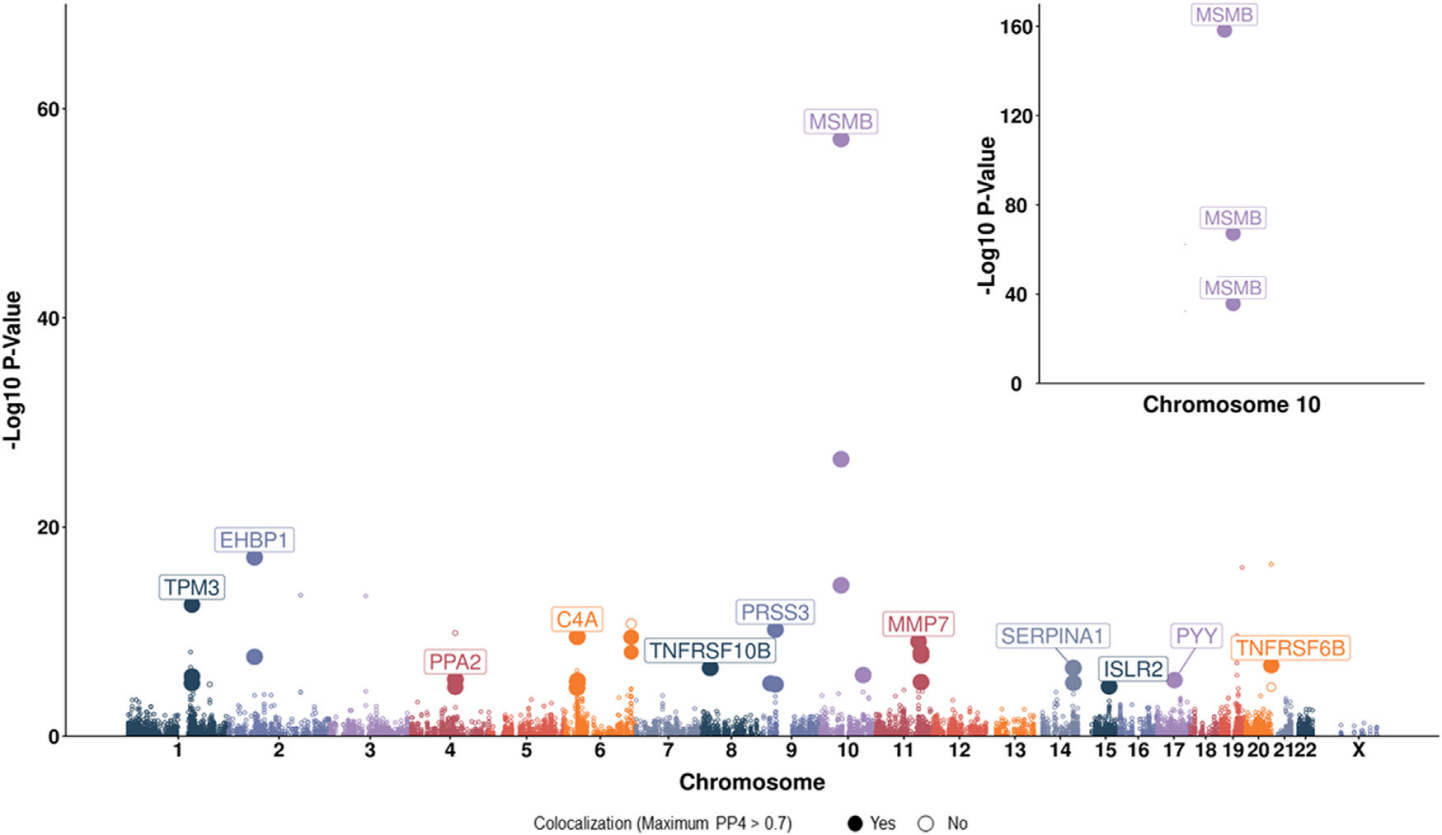
Association of genetically predicted protein concentrations with prostate cancer risk presented as a Manhattan plot where position is given by *cis-*pQTL coordinate (chromosome and base-pair position) labelled with their association with cancer risk and the highest colocalisation probability from single or conditional iterative methods (PP4). Points highlighted as filled-in are those with evidence of a shared causal locus (PP4 > 0.7) with point size reflecting PP4 magnitude, which can vary between 0 and 1. Risk associations with MR p > Bonferroni correction threshold were not subject to colocalisation analyses. The strongest protein-cancer association per chromosome is labelled and a zoomed-in plot for MSMB (rs10993994) on chromosome 10 is shown in the upper right-hand corner.

**Fig. 2:**
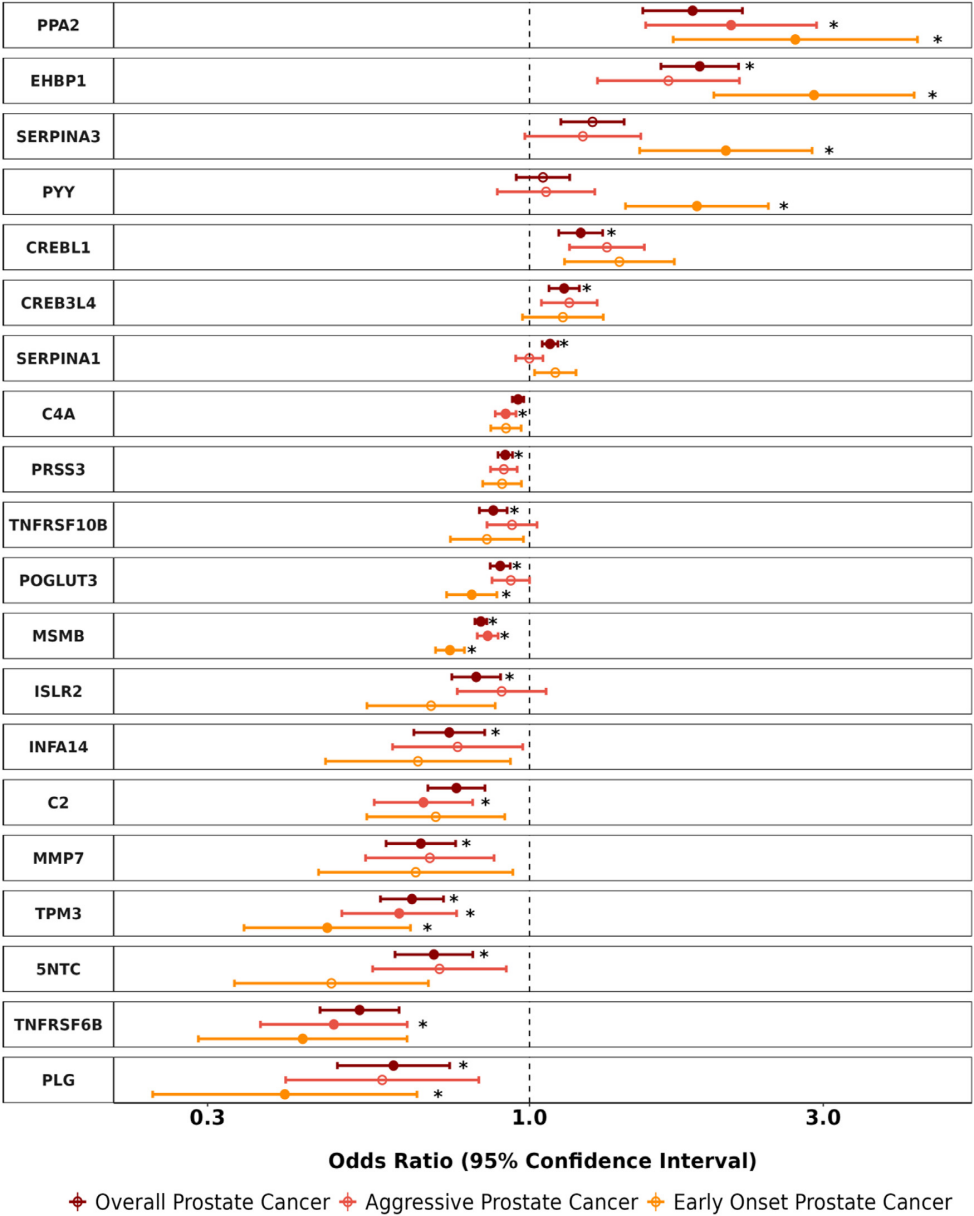
Odds ratios (95% confidence intervals) for genetically predicted protein levels and prostate cancer risk (for proteins with p < Bonferroni threshold based on 0.05/number of proteins analysed). Odds ratio estimates are scaled per standard deviation increment in genetically predicted relative circulating protein concentrations. Filled circles represent Bonferroni-significant associations and asterisks indicate evidence for colocalisation (PP4 > 0.70).

We investigated the associations of 2002 unique proteins using 4592 *cis*-pQTL that harmonised with the GWAS summary statistics for at least one of overall (1999 proteins; 4582 *cis*-pQTL), aggressive (1986 proteins; 4543 *cis*-pQTL), or early onset prostate cancer (1984 proteins; 4534 *cis*-pQTL) (Fig. 1). From these analyses we identified 20 proteins that were associated, after correction for multiple testing, with at least one outcome of overall (14 proteins), aggressive (7 proteins), or early-onset (8 proteins) prostate cancer and that also had support from colocalisation analyses (Fig. 2, Table 1). Of the 20 proteins associated with any prostate cancer outcome, several showed robust associations in only one outcome, including seven that appeared specific to overall prostate cancer (5NTC, CREBL1, INFA14, ISLR2, MMP7, SERPINA1, TNSFRS10B), three that appeared specific to aggressive disease (C4A, C2, TNFRSF6B), and two that appeared specific to early onset disease (SERPINA3, PYY).

**Table 1:** Mendelian randomisation and colocalisation results for protein-cancer associations that passed Bonferroni correction (0.05/n proteins analyzed) and colocalised at PP4 >0.70.

Prostate cancer outcome	Gene	SNP	Proportion variance explained (%)	Uniprot ID	Platform	Odds ratio (95% CI) practical	p-value (unadjusted)	Maximum PP4	Odds ratio (95% CI) UKBB/Finn gen	Odds ratio (95% CI) African ancestry	Drug target	Current drug trials
Overall	MSMB	rs10993994	49.6%	P08118	SomaScan	0.81 (0.8–0.82)	1.32E-165	1.00	0.83 (0.81–0.85)	0.85 (0.80–0.91)		Phase I (Prostate Cancer therapy; PCK-3145)
	EHBP1	rs73934251	0.53%	Q8NDI1	SomaScan	1.89 (1.63–2.19)	8.09E-18	0.98	2.09 (1.66–2.65)			
	TPM3	rs72696208	0.68%	P06753	SomaScan	0.64 (0.57–0.72)	2.72E-13	0.94	0.64 (0.53–0.77)		Phenethyl Isothiocyanate	
	PRSS3	rs2005617	14.5%	P35030	SomaScan	0.91 (0.89–0.94)	6.47E-11	1.00	0.95 (0.91–0.99)	0.95 (0.87–1.1)	4-(1,3,2-DIOXABOROLAN-2-YLOXY)BUTAN-1-AMINIUM; 4-HYDROXYBUTAN-1-AMINIUM; 4-(HYDROXYMETHYL)BENZAMIDINE; BENZAMIDINE; DIAMINO-N-[3-(1,3,2-DIOXABOROLAN-2-YLOXY)PROPYL]METHANIMINIUM; GUANIDINE-3-PROPANOL; [4-(1,3,2-DIOXABOROLAN-2-YLOXY)METHYL]BENZAMIDINE; 1,3,2-DIOXABOROLAN-2-OL	Biological testing
	PLG	rs982403	0.23%	P00747	SomaScan	0.48 (0.38–0.60)	2.96E-10	1.00	0.46 (0.31–67)		Tranexamic Acid	11 Drugs Launched
		rs11751347	1.66%	P00747	SomaScan	0.8 (0.75–0.86)	3.47E-10	0.83	0.84 (0.77–0.93)			
		rs4252185	0.23%	P00747	SomaScan	0.60 (0.49–0.74)	2.07E-06	0.99	0.60 (0.44–0.81)			
		IVW	2.11%			0.75 (0.62–0.92)	5.80E-03					
	MMP7	rs14983	1.56%	P09237	SomaScan	0.67 (0.58–0.76)	8.84E-10	0.98	0.62 (0.51–0.74)	0.83 (0.73–0.95)	Marimastat	1 in Phase I/II (Colorectal Cancer therapy; IMA-910)
	POGLUT3	rs74911261	9.89%	Q7Z4H8	SomaScan	0.90 (0.86–0.93)	9.99E-08	1.00	0.85 (0.80–0.91)	0.97 (0.87–1.1)		
	TNSFRS10B	rs2293400	4.30%	O14763	OLINK	0.87 (0.83–0.92)	3.02E-7	1.00	0.73 (0.68–0.79)		HGS-TR2J; Lexatumumab	4 in Phase II (Therapy for multiple cancer types)
	SERPINA1	rs28929474	16.10%	P01009	SomaScan	1.08 (1.05–1.11)	3.01E-07	1.00	1.05 (1.0–1.10)	1.06 (0.97–1.15)	Glassia	1 in Phase II/III (Alpha-1 antitrypsin deficiency; fazirsiran)
	5NTC	rs4919682	0.50%	P49902	SomaScan	0.70 (0.6–0.81)	1.43E-06	1.00	0.64 (0.49–0.84)			1 in Phase I (Prostate Cancer therapy; FP-253)
	CREBL1	rs8111	1.31%	Q99941	SomaScan	1.21 (1.12–1.31)	4.92E-06	0.99	1.23 (1.03–1.48)			
	CREB3L4	rs4845586	3.12%	Q8TEY5	SomaScan	1.14 (1.08–1.2)	7.71E-06	0.73	1.23 (1.13–1.34)			
	INFA14	rs662463	0.558%	P01570	SomaScan	0.74 (0.65–0.85)	9.04E-06	0.95	0.80 (0.64–0.99)			Biological Testing
	ISLR2	rs751527	1.36%	Q6UXK2	SomaScan	0.82 (0.75–0.90)	1.78E-05	0.97	0.84 (0.73–0.97)	0.85 (0.73–0.99)		
Early onset	MSMB	rs10993994	49.6%	P08118	SomaScan	0.71 (0.68–0.74)	1.46E-65	1.00				Phase I (Prostate Cancer therapy; PCK-3145)
	PLG	rs11751347	3.57%	P00747	SomaScan	0.63 (0.53–0.73)	9.38E-09	0.72				11 Drugs Launched
		rs11751347	1.66%	P00747	SomaScan	0.61 (0.52–0.72)	9.38E-09	0.72				
		rs982403	0.23%	P00747	SomaScan	0.26 (0.14–0.46)	4.07E-06	1.00				
		IVW				0.57 (0.36–0.91)	1.60E-02					
	EHBP1	rs73934251	0.53%	Q8NDI1	SomaScan	2.90 (2.0–4.2)	2.60E-08	0.98				
	TPM3	rs72696208	0.68%	P06753	SomaScan	0.47 (0.34–0.64)	1.92E-06	0.95			Phenethyl Isothiocyanate	1 Drug discontinued (Neurological Cancer therapy; anisina)
	PYY	rs8074783	0.0016%	P10082	SomaScan	1.87 (1.43–2.44)	4.36E-06	0.98				Preclinical
	POGLUT3	rs74911261	3.46%	Q7Z4H8	SomaScan	0.81 (0.73–0.89)	6.67E-06	1.00				
	SERPINA3	rs8023057	7.95%	P01011	SomaScan	2.08 (1.51–2.88)	8.04E-06	0.99			Zinc; Acetate, Chloride, Sulfate	Preclinical
	PPA2	rs4699179	0.378%	Q9H2U2	SomaScan	2.7 (1.71–4.27)	1.99E-05	0.93				
Aggressive	MSMB	rs10993994	49.6%	P08118	SomaScan	0.84 (0.82–0.86)	1.55E-35	1.00				Phase I (Prostate Cancer therapy; PCK-3145)
	TNFRSF6B	rs6011040	0.50%	O95407	SomaScan	0.48 (0.37–0.63)	1.72E-07	0.9				
	PPA2	rs4699179	0.38%	Q9H2U2	SomaScan	2.13 (1.54–2.93)	3.73E-06	0.99				
	C4A	rs2763982	22.0%	P0C0L4	SomaScan	0.91 (0.88–0.95)	6.80E-06	0.99				Preclinical
	TPM3	rs72696208	0.68%	P06753	SomaScan	0.61 (0.50–0.76)	8.56E-06	0.96			Phenethyl Isothiocyanate	
	PRSS3	rs62555900	3.52%	P35030	SomaScan	0.80 (0.73–0.89)	1.13E-05	0.99			4-(1,3,2-DIOXABOROLAN-2-YLOXY)BUTAN-1-AMINIUM; 4-HYDROXYBUTAN-1-AMINIUM; 4-(HYDROXYMETHYL)BENZAMIDINE; BENZAMIDINE; DIAMINO-N-[3-(1,3,2-DIOXABOROLAN-2-YLOXY)PROPYL]METHANIMINIUM; GUANIDINE-3-PROPANOL; [4-(1,3,2-DIOXABOROLAN-2-YLOXY)METHYL]BENZAMIDINE; 1,3,2-DIOXABOROLAN-2-OL	Biological testing
	C2	rs3094662	0.75%	P06681	SomaScan	0.67 (0.56–0.81)	2.39E-05	0.96				Phase III (Cancer immunotherapy; 99mTc-ior C5)

Results are shown per SNP-cancer association, except for those proteins for which there were multiple SNPs that passed both multiple testing correction and colocalisation, and for which the summary estimate using the Inverse Variance Weighted (IVW) method is provided. Odds ratios (95% confidence intervals) are oriented per standard deviation increase in genetically predicted protein level. Maximum PP4 is reported as highest PP4 value from either single or conditional iterative colocalisation method assessing the probability of a shared causal locus. Odds ratios (95% confidence intervals) are reported for associations using external UK Biobank/FinnGen and for an African ancestry population where data existed. Drug targets and drug trials are annotated where they existed.

The most statistically significant associations per standard deviation increase in protein level, with evidence of colocalisation were seen for MSMB (a protein that is specifically expressed in the prostate) with a lower risk of all prostate cancer endpoints [OR_Overall_ = 0.81, 95% CI: 0.79–0.82, PP4: 100%; OR_Aggressive_ = 0.84, 95% CI: 0.82–0.86, PP4: 0.99; OR_Early Onset_ = 0.71, 95% CI: 0.68–0.74, PP4: 1.0, Table 1, Fig. 2]. TPM3 was the only other protein that had a significant, colocalised association with risk of all outcomes [OR_Overall_ = 0.64, 95% CI: 0.57–0.73, PP4: 0.94; OR_Aggressive_ = 0.61, 95% CI: 0.49–0.76, PP4: 0.96; OR_Early Onset_ = 0.47, 95% CI: 0.34–0.64, PP4: 0.95, Fig. 2].

We also reported proteins with a colocalised association for one outcome and little evidence for an association with others after correction for multiple testing, such as IFNA14, ISLR2, MMP7, and TNSFRS10B which were associated exclusively with overall prostate cancer [OR_IFNA14_ = 0.74, 95% CI: 0.70–0.78; OR_ISLR2_ = 0.82, 95% CI: 0.75–0.90; OR_MMP7_ = 0.67, 95% CI: 0.58–0.76; OR_TNFRSf10B_ = 0.87, 95% CI: 0.83–0.92, Fig. 2]. Similarly, PYY and SERPINA3 associated with an increased risk of early onset prostate cancer only [OR_PYY_ = 1.87, 95% CI: 1.43–2.44; OR_SERPINA3_ = 2.08, 95% CI: 1.51–2.88, Fig. 2] while C2 associated with aggressive prostate cancer only [OR_C2_ = 0.67, 95% CI: 0.56–0.81, Fig. 2].

We additionally identified proteins with evidence for a directionally concordant and colocalised association with some, but not all, prostate cancer outcomes, including TNFRSF6B, which had an inverse association with all outcomes but only showed evidence in favour of colocalisation for aggressive disease [OR_Overall_ = 0.53, 95% CI: 0.46–0.61, PP4: 0.00; OR_Aggressive_ = 0.48, 95% CI: 0.0.37–0.63, PP4: 0.90; OR_Early Onset_ = 0.43, 95% CI: 0.29–0.63, PP4: 0.14, Fig. 2, Supplementary Table S3]. Likewise, PPA2 was associated with an increased risk of both aggressive and early onset disease, but lacked support from colocalisation analyses for prostate cancer risk overall [OR_Overall_ = 1.84, 95% CI: 1.52–2.22, PP4: 0.01; OR_Aggressive_ = 2.13, 95% CI: 1.54–2.93, PP4: 0.99; OR_Early Onset_ = 2.70, 95% CI: 1.71–4.27, PP4: 0.93, Fig. 2, Supplementary Table S3].

### Replication of robust proteins in European and African ancestry populations

**Fig. 3:**
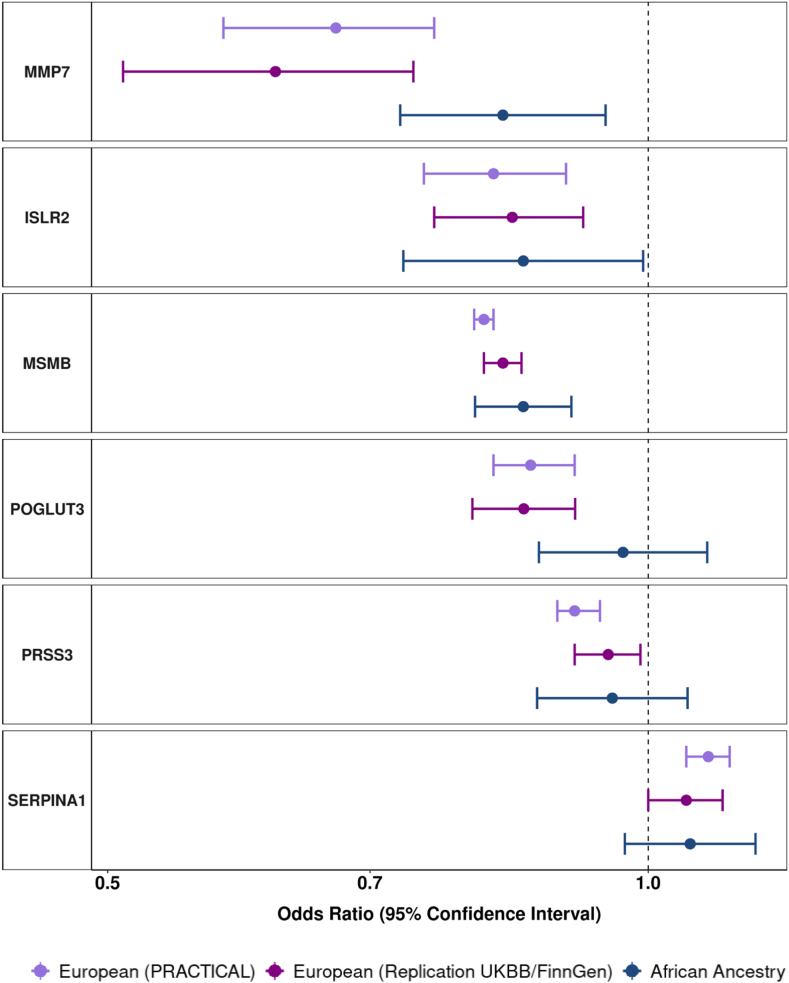
Odds ratios (95% confidence intervals) for genetically predicted protein levels and overall prostate cancer risk for proteins with p < Bonferroni threshold based on 0.05/number of proteins analysed in main analyses, and with data available to perform replication in an African ancestry and European ancestry population (UKBB = UK Biobank). Odds ratio estimates are scaled per standard deviation increment in genetically predicted circulating protein concentrations.

We replicated the association for all 14 proteins that were robustly associated with overall prostate cancer (5NTC, CREBL1, CREB3L4, EHBP1, INFA14, ISLR2, MMP7, MSMB, PRSS3, PLG, POGLUT3, SERPINA1, TNSFRF10B, TPM3) using an independent meta-analysis of European ancestry participants in the UK Biobank and FinnGen cohorts (Table 1). Among these, the most statistically significant association was for MSMB [OR_Overall_ = 0.83, 95% CI: 0.81–0.85, Table 1] and the largest effect size was for PLG [OR_Overall_ = 0.46, 95% CI: 0.07–0.84, Table 1]. We additionally identified African ancestry-specific *cis-*pQTL for six of the 14 proteins (ISLR2, MMP7, MSMB, POGLUT3, PRSS3, SERPINA1; Table 1). Of these, three protein associations with risk of prostate cancer overall were replicated in men of African ancestry: MSMB [OR_Overall_ = 0.85, 95% CI: 0.80–0.91], MMP7 [OR_Overall_ = 0.83, 95% CI: 0.73–0.95], and ISLR2 [OR_Overall_ = 0.85, 95% CI: 0.73–0.99] (Fig. 3, Table 1).

### Drug target analysis

Out of the 2002 unique proteins investigated, we identified 525 proteins that could be successfully mapped at the gene level to the target of a therapeutic intervention. Of these, ten (TPM3, PRSS3, PLG, MMP7, SERPINA1, SERPINA3, TNFRSF10B, C4A, HDGF, and LAYN) were associated with risk of at least one prostate cancer outcome after correction for multiple testing (525 proteins), and also showed evidence of colocalisation (Supplementary Table S4). For example, PLG and C4A mapped to the clot dissolving class of fibrinolytics, TPM3 mapped to phenethyl isothiocyanate, and MMP7 mapped to matrix metalloproteinase inhibitor, marimastat.

### Spatial transcriptomic analysis

**Fig. 4:**
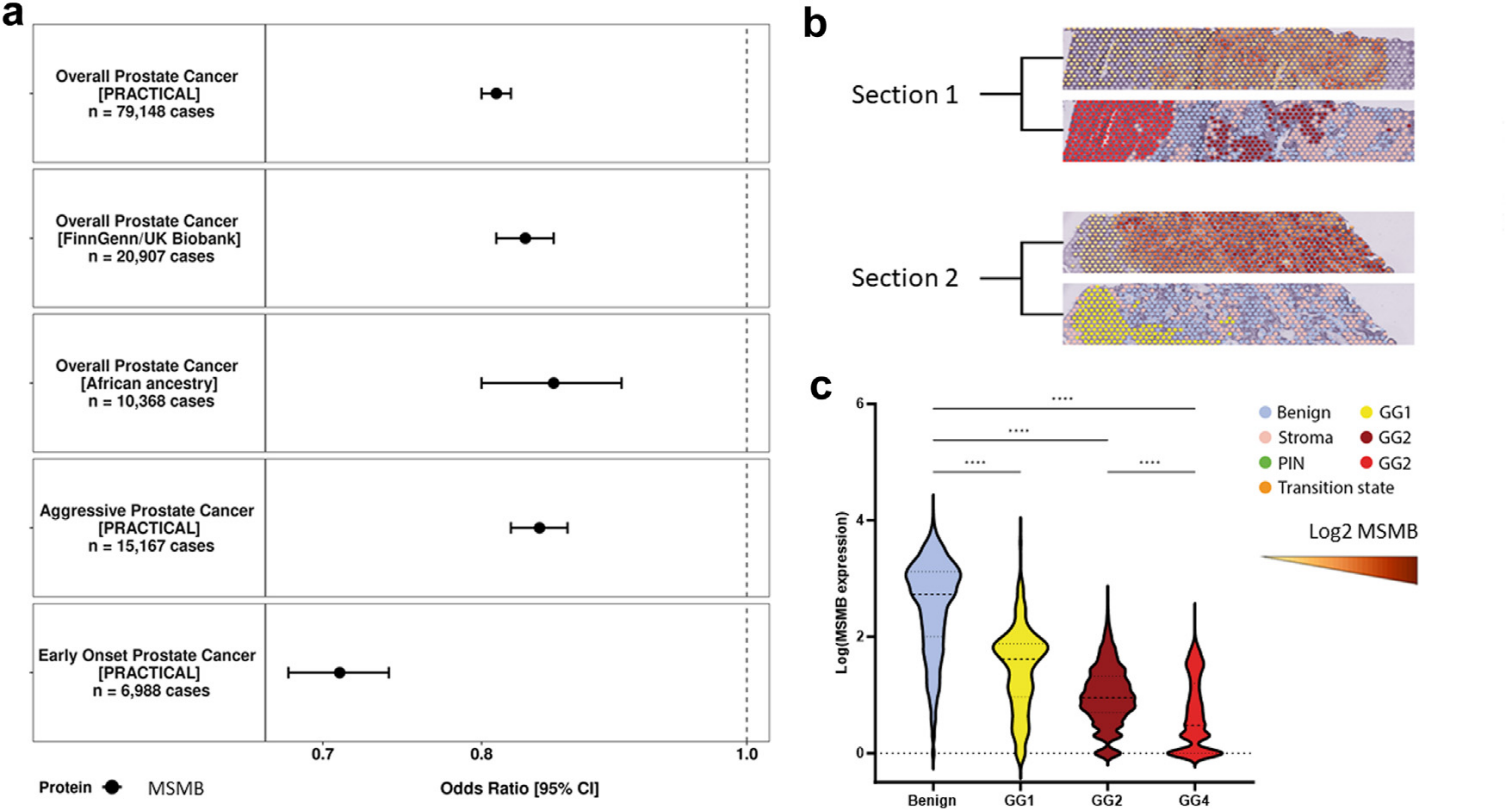
**a)** MSMB association with overall, early onset, and aggressive prostate cancer risk with replication in the FinnGen and UK Biobank populations and in an African ancestry population. Odds ratio (95% confidence interval) estimates are scaled per standard deviation increment in genetically predicted circulating MSMB concentrations **b)** Spatial visualisation showing MSMB gene expression (top) and histology and tissue status (bottom) from organ-wide spatial transcriptomic data in two tumour sections (GG: Gleason grade group: GG1, Gleason score of 6 or lower; GG2, Gleason score of 3 + 4 = 7; GG4, Gleason score of 8). **c)** Violin plots representing gene expression in each spatial transcriptomics spot according to histological status. Statistical differences are indicated: ∗∗∗∗p < 0.0001 (Kruskal–Wallis; post-test: Dunn's test).

**Fig. 5:**
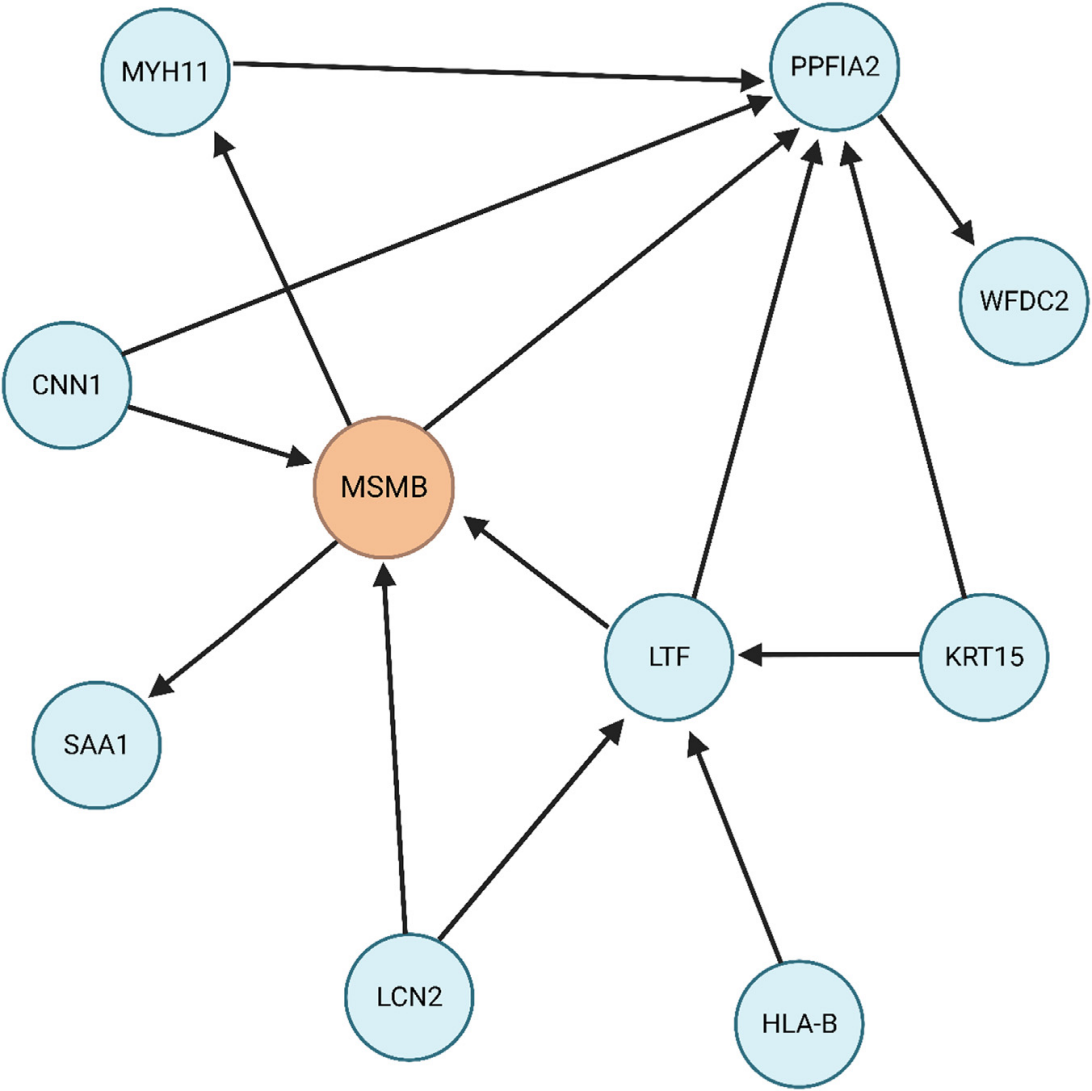
Gene network from iterative random forests of the difference in gene expression between benign and GG4 prostate histology (Gleason Score = 8). Arrows indicate direction of influence and shape of the network. MSMB is coloured to demonstrate its central role in the network.

Of the 20 proteins that were associated with at least one prostate cancer outcome, we performed a targeted follow-up analysis for the two proteins with known high expression in the prostate tumour epithelium, MSMB and CREB3L4, using organ-wide spatial transcriptomic data on tissue obtained by radical prostatectomy from a patient with multifocal prostate cancer.^35^ In analysing epithelial-rich spots, we observed marked differences of MSMB expression between benign cells, where MSMB was highly abundant, and Gleason grade group 4 (GG4) cells, where MSMB was very low or absent in a majority of cells (*RNA Count* MSMB_*benign*_ [median, interquartile range]: 532 [97–1310] vs. MSMB_*GG4*_: 1.0 [0.0–6.0], Fig. 4c). A similarly, albeit more modestly, lower MSMB expression was observed in GG1 and GG2 cells (MSMB_*GG1*_: 40.0 [8.0–75.0] and MSMB_*GG2*_: 9.0 [4.0–21.0] compared to benign cells (Fig. 4c). A lower expression of CREB3L4 was noted in GG2 and GG4 cells compared to benign cells CREB3L4*_benign_*: 3.0 [1.0–6.0] vs. CREB3L4_*GG2*_: 1.0 [0.0–2.0] vs. CREB3L4_*GG4*_: 0.0 [0.00–2.0], Supplementary Figure S2b). Additional genome-wide random forest analyses identified MSMB expression as the most important gene to distinguish between benign and GG4 cells (Fig. 5).

## Discussion

In this analysis, we conducted the largest study to date investigating the genetic associations of up to 2002 unique proteins with the likely aetiological risk of overall, early onset, and aggressive prostate cancer in up to 177,526 men using a MR and colocalisation pipeline. In total, we found evidence supporting associations between 20 proteins and prostate cancer risk: 14 proteins for overall prostate cancer risk, seven for aggressive prostate cancer and eight for early onset disease. Among those 14 proteins that associated with prostate cancer risk overall, 14 were replicated in an external European ancestry population and three (out of six with available data) were replicated in an African ancestry population. A further half of the 20 proteins identified were also found to be the site of action for established drug targets with potential therapeutic implications. Finally, using spatial transcriptomics data, we demonstrated a central role for the gene of our most robustly associated prostate cancer protein, MSMB, in distinguishing benign from undifferentiated, high-grade prostate cancer cells.

### MSMB and integrated evidence of prostate cancer risk

In this study, MSMB was one of only two proteins (including TPM3) to significantly associate and colocalise with all three prostate cancer outcomes. MSMB is a secretory protein and member of the human immunoglobulin family that is released largely by luminal epithelial cells in the prostate epithelium and has a documented role in overall prostate cancer risk for both observational and genetic epidemiology.^6^^,^^7^^,^^37^ In this study, we expand upon these previous findings by demonstrating that higher levels of genetically predicted MSMB are associated with a 20% lower risk of overall prostate cancer in two independent European ancestry cancer GWAS, and confirm its protective role in both aggressive and early onset disease aetiology. We further successfully replicated this association with prostate cancer risk overall in an African-ancestry population, an ancestry group with an established higher risk of developing the disease. Subsequently, in reporting that MSMB gene expression is significantly depleted among high grade tumour when compared to expression in benign cells, we reiterate, through an independent line of evidence, that this gene may be particularly relevant to tumourigenesis and risk for aggressive disease.

Although the mechanism of action for MSMB in prostate cancer is not clear, MSMB has been shown to have a regulatory effect on cell growth, which may be lost during tumourigenesis while a MSMB-derived polypeptide was shown to induce prostate cell death.^37^^,^^38^ Additionally, in a rodent model and *in vitro*, higher MSMB activity was found to suppress prostate tumour growth while a knockout of MSMB promoter/enhancer regions was characterized by tumour progression and metastases.^39^^,^^40^ Given the integration of several compelling lines of evidence presented in this paper with existing literature, further research is warranted to understand the precise functional role of MSMB in prostate cancer tumourigenesis, identify environmental and lifestyle determinants, and explore its potential clinical utility.

### CREB3L4 and CREBL1 and risk of prostate cancer overall

We identified 14 proteins associated with overall prostate cancer aetiology. These include the endoplasmic reticulum (ER) originating transcription factors CREB3L4 and CREBL1 (also known as ATF6B), which we find are associated with an 14% and 21% increased risk of prostate cancer overall, respectively, and that are expressed in the prostate epithelium.^34^ These transcription factors form part of a transcriptional factor network that regulates the function of the endoplasmic reticulum (ER) and the activity of the unfolded protein response (UPR). There is an established role for the UPR and heat-shock proteins in maintaining androgen receptor (AR) stability and supporting AR-dependent tumourigenesis.^41^^,^^42^ CREB3L4 has been shown to directly interact with AR in LNCaP cells to increase cellular proliferation and is abundantly expressed in prostate tumour tissue.^43^^,^^44^ Furthermore, evidence suggests that disruptions in CREB3L4 contribute to ER stress downstream and initiation of the unfolded protein response.^44^^,^^45^ In a previous study of differential gene expression in prostate tissue, CREB3L4 was identified as a member of a co-expression gene cluster enriched for a previously described metabolic pathway (hsa05215) in prostate cancer that may regulate apoptosis and cell proliferation.^46^ Interestingly, we found that CREB3L4 expression is lower in GG2 and GG4 cells as opposed to benign cells.

Previous studies have linked AR activation with members of the ATF6 family in LNCaP and PC3 cells, however these experiments have mostly focused on ATF6A.^47^^,^^48^ For example, a recent *in vivo* study showed that prostate cancer cells with ATF6A overexpression resisted cellular death by ferroptosis.^47^ In parallel, a previous MR study reported a lowered risk of prostate cancer overall with genetically elevated circulating ATF6A levels from *trans-*pQTL.^49^ Given the promising role of its paralog, and the increased risk we report here, targeted follow up of CREBL1 may prove valuable in characterising the broader role of ER stress proteins and AR-dependent tumourigenesis.

### Other aggressive and early onset prostate cancer proteins

We additionally found several proteins that were associated with risk for either aggressive or early onset disease, including PRSS3, PPA2, EHBP1, PYY, C2 and C4A. PR33S, a member of the trypsin family, was associated with a 20% reduced odds of aggressive disease in our study. While one previous study found that mesotrypsin, a protease encoded by the PRSS3 gene, was essential for prostate cancer metastasis in vitro and mouse models, the role of this protein and other isoforms have not been widely studied in humans.^50^ Likewise, we found PPA2, a serine/threonine phosphatase which has been shown to account for up to 50% of global cellular phosphatase activity, was associated with a two-fold increased odds of aggressive disease. While few studies have directly assessed the relationship between PPA2 and risk of prostate cancer prospectively, a proteomic tissue analysis comparing men with localised prostate cancer to men with lymph node metastasised prostate cancer found significantly higher levels of PPA2 in men with the more advanced disease.^51^^,^^52^

We observed a more than two-fold increased risk of early-onset prostate cancer associated with higher EHBP1, an adaptor protein with a key role in vesicular trafficking and actin reorganization.^53^ Variants in the EHBP1 intron have previously been associated with aggressive prostate cancer in a genetic association study of Europeans and North Americans.^54^ The protein was also found to be expressed in prostate tumour tissue and may have a role in determining the invasiveness of PTEN-positive prostate cancer cells, according to GWAS and expression data from The Cancer Genome Atlas, and in a cellular study.^54^, ^55^, ^56^ While mechanisms that may link EHBP1 to prostate cancer risk are not yet fully described, it has a role as an effector molecule for Rab8 family members that modulate polarised membrane transport via actin reorganisation and may have a role in the mechanism of action for atorvastatin.^55^ Likewise, we found that PYY, a metabolic hormone involved in appetite regulation, was also associated with an increased odds of early onset disease in our study. While there has been some hypothesised relationship between obesity and aggressive prostate cancer risk in the past, recent findings suggest that obesity does not serve as a risk factor for disease itself, but rather may affect likelihood of diagnosis.^57^^,^^58^

We also note that proteins associated with early onset disease were generally greater in magnitude when compared to their associations with overall or aggressive disease. Two of these proteins, the complement proteins C2 and C4A, sit on chromosome six, which contains a particularly dense genetic region including the MHC complex and is consequently particularly difficult to interpret. However, given the importance of addressing early onset and aggressive disease, future studies are needed to further investigate and replicate the associations with early onset disease to uncover potential subtype specific mechanisms of disease onset and progression.

### Drug target proteins

We identified 10 proteins that were associated with both the risk of prostate cancer and that were the site of action for a known drug. These included TNFRSF10B, which is a receptor for the cytotoxic TRAIL ligand, and is essential for CASP8 and ER stress induced apoptosis.^59^ Further, TNFRSF10B expression is lower in higher grade prostate tumours and a recent study of PARP inhibitors in prostate cancer cell lines suggested that TNFRSF10B may provide a mechanism by which the cancer drug olaparib induces apoptosis.^60^ The apparent protective association we observe with prostate cancer risk is in line with the results from multiple phase I/II trials of TNFRSF10B agonists that support their use for the treatment of multiple cancer endpoints, though not yet including prostate cancer.^60^, ^61^, ^62^

We also identified an inverse association of PLG, a serine protease targeted by transexamic acids and several classes of thrombolytics, with prostate cancer risk overall and with early onset disease. Transexamic acids are primarily prescribed to control excessive bleeding while thrombolytics are primarily used to dissolve blood clots and act via plasmin and fibrin pathways.^63^ One molecular study found that PLG is generated by the cancer-mediated proteolysis of plasminogen which is released by human prostate carcinoma cells.^64^ PLG in turn has been shown in many lab studies to inhibit angiogenesis which when unregulated can lead to the rapid formation of tumours.^65^^,^^66^ Currently, several studies are investigating combination therapy including plasminogen activation or inhibition for treatment of several cancer types, though not specifically for prostate cancer, in phase I/II trials.^67^^,^^68^

SERPINA1 maps to fazisiran, the treatment for alpha-1 antitrypsin deficiency and that is in phase II/III of drug trials. Previous findings have indicated that alpha-1 antitrypsin levels are often elevated in many carcinomas, including prostate.^69^ However, no agents targeting SERPINA1 have been investigated in cancer trials thus far. MMP7 belongs to a class of matrix metalloproteinases that participate in wound healing, bone growth, and matrix remodelling. There are multiple lines of evidence that suggest this protein is involved in many cancers, and agents targeting metalloproteases, such as marimastat, are currently being investigated in clinical trials at various phases.^70^ In prostate cancer, marimastat showed some efficacy in early trials, however has not yet progressed further.^71^

While we highlight proteins that share the same target site for established drug targets that may have implications for therapeutic use, the suitability of these to act as preventative or remedial agents requires careful considerations including: site specificity, potential downstream effects, routes of administration and effectively capturing the population at risk.^72^

### Study strengths

This study offers several strengths including being the largest currently available GWAS of prostate cancer outcomes and the use of both aggressive and early onset endpoints with *cis-*pQTL covering up to 2002 proteins. One previous MR study investigated the role of the circulating proteome in prostate cancer risk but did not stratify analyses by *cis* or *trans-*pQTL, and did not perform colocalisation analyses, making it more challenging to infer causal relationships between individual proteins and cancer risk.^49^ Additionally, by integrating gene expression data measured using spatial transcriptomics data, the current paper introduces a translational approach to highlight biological enablers of prostate cancer. To our knowledge, this study provides the first demonstration that MR using *cis* instruments of plasma protein levels can be used to identify a risk protein that has both specific expression in the cell of cancer origin and is related to tumour aggressiveness—important features to consider when identifying candidate targets for therapeutic prevention.

### Caveats and limitations

While we have analysed a wide array of proteins, we have not investigated the entire human plasma proteome (n ∼ 20,000 protein-coding genes). As more protein GWAS data become available, it will become possible to use genetic methods to investigate more proteins. However, some blood proteins are unlikely to have a *cis-*pQTL due to the degree of evolutionary constraint for a protein's cognate gene. Additional limitations include the more modest GWAS sample sizes for aggressive and early onset prostate cancer, which have lower power to discover novel protein associations. Finally, while we were able to perform additional analyses to replicate some, but not all, of our robust proteins in populations of African ancestry, we note as a limitation that GWAS sample sizes in this group are not yet sufficient to perform well-powered discovery analyses. Especially given the increased risk for prostate cancer among populations of African ancestry, it is essential that future studies identify risk proteins in more diverse populations and allow for the discovery of ancestry-specific markers of risk.

### Conclusion

This paper provides a catalogue of 20 proteins with evidence of aetiological significance for prostate cancer. These proteins present an opportunity to direct further molecular and epidemiological investigations aimed at exploring the specific roles that the proteome plays in tumourigenesis and ultimately may inform future research into therapeutic prevention. In particular, converging evidence from population genetic and tumour sequencing analyses implicates MSMB as having an important protective role in prostate tumourigenesis, both in European and African-ancestry men, which is particularly marked for aggressive and early onset disease.

## Contributors

All authors have read and approved the final version of the manuscript. TAD and KSB accessed and verified the underlying data. Authors contributed as following:

**Conceptualization:** RCT, KSB, TJK, TAD, AKH, MD.

**Data Curation:** PRACTICAL Consortia, KSB, AKH, JRT, CL, MP, MK, AM, ADL, SF, WY.

**Formal Analysis:** TAD, SF, WY, MD, AVS.

**Funding Acquisition:** RCT, TJK.

**Methodology:** KSB, RCT, TD.

**Supervision:** KSB, RCT, TJK, ELW.

**Writing Original Draft:** TAD, KSB.

**Reviewing and Editing:** All authors: TAD, AKH, MD, SF, WY, ELW, JRT, AVS, HBS, KKT, MJG, RMM, MJ, MP, CL, IGM, ADL, AM, TJK, RCT, KSB.

Data used in this study were generated by the PRACTICAL Consortium and all affiliated members were invited to review the final manuscript.

## Data sharing statement

PQTL data were obtained through publicly available GWAS. Summary statistics from Zheng et al. (2020) can be obtained from OpenGWAS (https://gwas.mrcieu.ac.uk/), from Folkersen et al. (2020) at http://www.scallop-consortium.com, from Ferkingstad et al. (2021) at https://www.decode.com/summarydata/, and from Pietzner et al. (2021) at https://omicscience.org. Summary statistics for overall prostate cancer were available through the PRACTICAL Consortium (http://practical.icr.ac.uk/?page_id=8164) and approval was received to obtain summary statistics for further cases using [dbGaP] project #31553, and for aggressive and early onset disease from the PRACTICAL Consortium (for enquiries contact PRACTICAL@icr.ac.uk).

## Declaration of interests

This work was supported by Cancer Research UK (grant no. C8221/A29017).

Anders Mälarstig, Åsa Hedman, and Marios Dimitriou are employees of Pfizer Inc. Anders Mälarstig declares stock options for Pfizer Inc. Alastair D. Lamb is Section Editor for Prostate Cancer and Web, British Journal of Urology International.
