# Supplementary material for: Identifying proteomic risk factors for overall, aggressive, and early onset prostate cancer using Mendelian Randomisation and tumour spatial transcriptomics
Source: eBioMedicine. 2024 Jun 14;105:105168. doi: 10.1016/j.ebiom.2024.105168 (PMC11233900; doi:10.1016/j.ebiom.2024.105168)
Supplement: Supplementary material [file mmc2.docx]

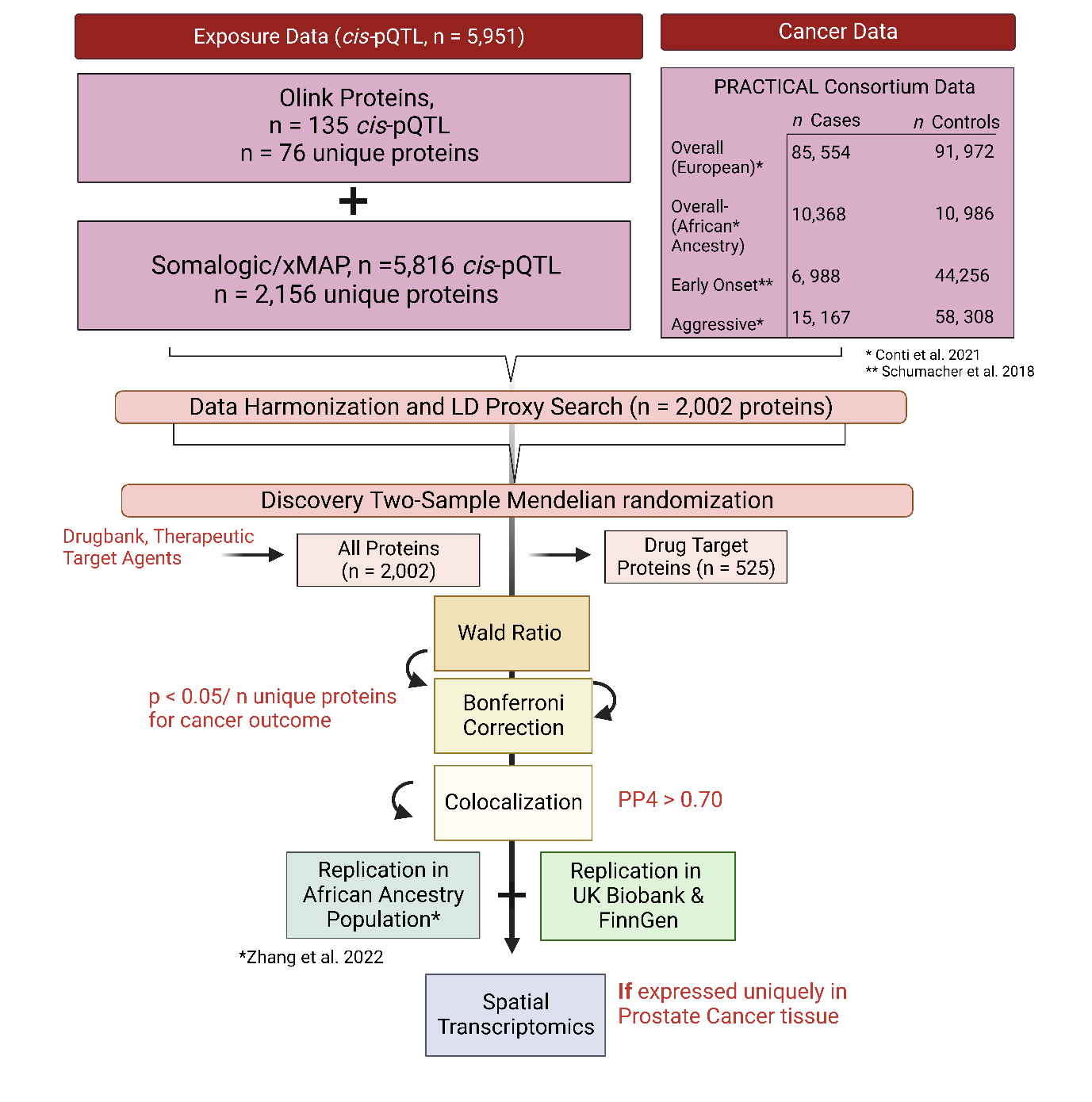


**Supplementary Figure 1. Flow-chart showing the overall study design**. PP4 = posterior probability of a shared causal locus. LD = Linkage disequilibrium.


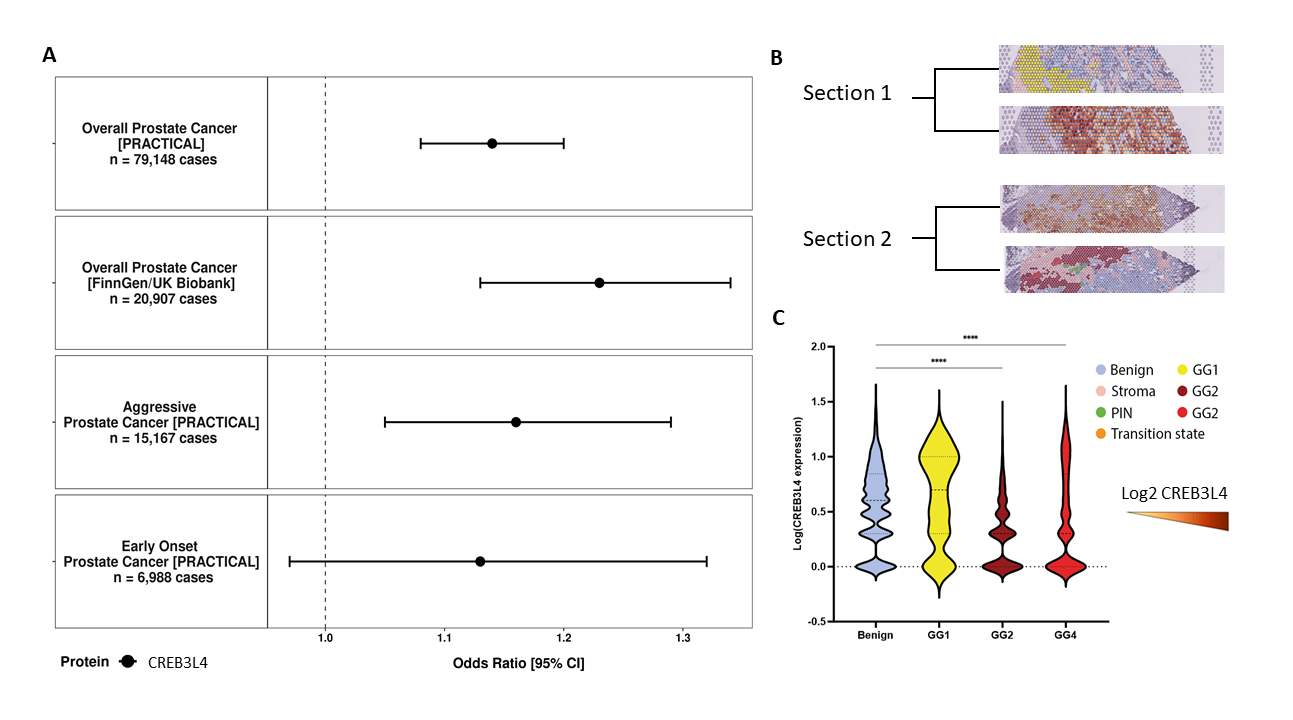


**Supplementary Figure 2. a)** CREB3L4 association with overall, early onset, and aggressive prostate cancer risk with replication in the FinnGen and UK Biobank populations. Odds ratio (95% confidence interval) estimates are scaled per standard deviation increment in genetically predicted circulating CREB3L4 concentrations **b)** Spatial visualization showing CREB3L4 gene expression (top) and histology and tissue status (bottom) from organ-wide spatial transcriptomic data in two tumour sections (GG: Gleason grade group: GG1, Gleason score of 6 or lower; GG2, Gleason score of 3+4 = 7; GG4, Gleason score of 8). **c)** Violin plots representing gene expression in each spatial transcriptomics spot according to histological status. Statistical differences are indicated: **** p < 0.0001 (Kruskal–Wallis; post-test: Dunn’s test).

**Supplementary Table 1.** Descriptive study characteristics from which underlying pQTL data were extracted.

**Supplementary Table 2.** Full protein exposure data for all instruments used in the study, before quality control and harmonisation. N = study sample size.

**Supplementary Table 3.** Mendelian randomisation and colocalisation results for all proteins and cancer outcomes, where analysed. Odds ratios (95% confidence intervals) are given per standard deviation increase in genetically predicted protein level, and maximum colocalisation indicates the highest PP4 percentage from either single or conditional iterative colocalisation methods.

**Supplementary Table 4.** Results for proteins identified as robust in either main analysis or drug target analyses with mapping to drug targets and highest level of therapeutic investigation. Odds ratios (95% confidence intervals) are given per standard deviation increase in genetically predicted protein level, and maximum colocalisation indicates the highest PP4 percentage from either single or conditional iterative colocalisation methods.
